# Association Between the Pretreatment Albumin-to-Alkaline Phosphatase Ratio and Clinical Outcomes in Patients With Bladder Cancer Treated With Radical Cystectomy: A Retrospective Cohort Study

**DOI:** 10.3389/fonc.2021.664392

**Published:** 2021-04-20

**Authors:** Shijie Li, Shiyang Lu, Xuefeng Liu, Xiaonan Chen

**Affiliations:** Department of Urology, Shengjing Hospital of China Medical University, Shenyang, China

**Keywords:** bladder cancer, radical cystectomy, albumin-to-alkaline phosphatase ratio, prognostic impact, nomogram

## Abstract

**Objective:**

Serum albumin-to-alkaline phosphatase ratio (AAPR) has been proven to be a prognostic indicator of many malignant tumors. However, whether it can predict the prognosis of bladder cancer (BC) patients who underwent radical cystectomy (RC) remains unclear. This study was designed to assess the relationship between AAPR and clinical outcomes in patients with BC treated with RC.

**Methods:**

The clinicopathological data of 199 BC patients receiving RC in our institution from January 2012 to December 2017 were retrospectively collected and analyzed. They were divided into three groups based on the optimal cut-off values and the association between AAPR groups and their clinical outcomes were evaluated.

**Results:**

The average age of the patients was (64.0 ± 8.7) years and 79.9% were male. Based on the cut-off values of AAPR, patients were divided into three groups: low-AAPR group (AAPR < 0.37, n = 35), medium-AAPR group (AAPR = 0.37-0.59, n = 61) and high-AAPR group (AAPR > 0.59, n = 103). The median overall survival (OS) of each AAPR group was 12.5, 24, and 29 months, respectively (*P* value <0.0001). After adjusting the Cox proportional hazards model, medium- and high- AAPR groups showed a reduced risk trend of death, with a risk ratio of 0.44 (95% CI = 0.21-0.91) and 0.25 (95% CI = 0.12-0.49), respectively (*P* for trend <0.001). No nonlinear relationship was identified by smooth fitting curve between AAPR and OS. By subgroup analysis, we observed that compared to the low-AAPR group, the trends of the HRs in the medium- and high-AAPR group were decreased across nearly all subgroups after stratification. Moreover, the AAPR-based nomograms for OS, CSS and RFS were also constructed. The C-index showed a good predictive accuracy (OS, C-index 0.728, 95% CI 0.663-0.793; CSS, C-index 0.792, 95% CI 0.748-0.838; RFS, C-index 0.784, 95% CI 0.739-0.829).

**Conclusion:**

Pretreatment AAPR is significantly associated with the prognosis of BC patients receiving RC, which can be conducive to the clinical decision-making and risk stratification in those patients. The nomogram based on AAPR is a reliable model for predicting survival of BC patients after RC.

## Introduction

Bladder cancer (BC) is one of the most prevalent urological cancers all around the world, with an increasing rate of morbidity and mortality (1). In China, it is the most commonly occurring genitourinary malignant tumor and is one of the main causes of cancer-related deaths (2). According to the depth of tumor infiltration into the bladder wall, BC can be categorized into two types: non-muscle invasive bladder cancer (NMIBC) and muscle invasive bladder cancer (MIBC), with about 25% of BC being MIBC at first diagnosis. Local or distant metastases occur in 15% of patients; in addition, approximately 30% to 40% of MIBC cases develop lymph node or distant metastases during the course of the disease (3). Although radical cystectomy (RC) produces relatively long-term survival rates in high-risk BC patients, the risk of local recurrence and distant metastasis is high and the prognosis is nevertheless poor in these patients (4). About 50% of high-risk patients will have distant metastasis and the 5-year survival rate is only 40%-60% (5).

Some clinicopathological characteristics have been shown to be closely associated with adverse outcomes, such as advanced stage (6), lymph node metastasis (7), poor differentiation (8), and anemia (9). However, patients with similar features to one another often have different clinical outcomes. Moreover, in most cases, precise information about these prognostic features, such as lymph node metastasis and the accurate pathological stage and grade of patients, can only be accurately diagnosed after surgery. Therefore, it is necessary to assess the prognosis of BC patients who underwent RC preoperatively.

Many preoperative blood-based markers, such as albumin(ALB) (10), C-reactive protein (11), neutrophil lymphocyte ratio (12), platelet lymphocyte ratio (13), and lymphocyte monocyte ratio (14), have been shown to be prognostic factors in bladder cancer. In our study, we combined two laboratory parameters: ALB with alkaline phosphatase (ALP) to create a novel function-based serum biomarker called albumin-to-alkaline phosphatase ratio (AAPR). Serum AAPR is a comprehensive reflection of a host’s nutritional status, which has been widely proved to have predictive value for multiple cancers, including renal cell carcinoma, small cell lung cancer, nasopharyngeal carcinoma and hepatocellular carcinoma (14–18). However, to the best of our knowledge, it has not yet been studied in BC patients undergoing RC. Therefore, we conducted a study aiming to confirm the potential prognostic impact of pretreatment AAPR in BC patients undergoing RC.

## Materials and Methods

### Patients Selection

**Figure 1:**
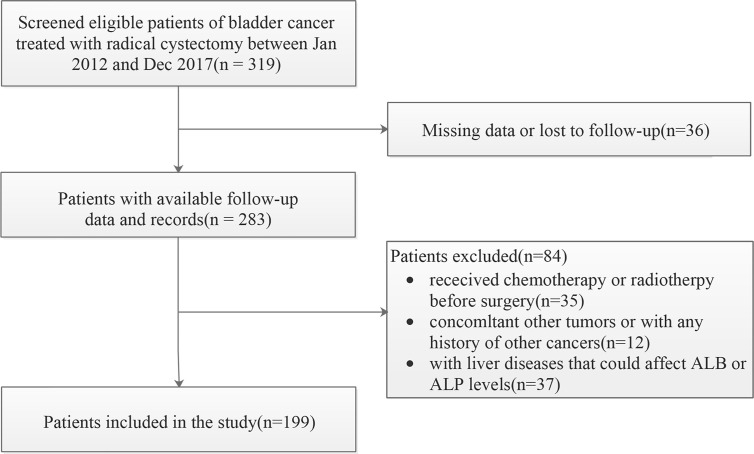
The patient selection flowchart.

We evaluated consecutive patients with BC who underwent RC in our institution from January 2012 to December 2017. The initial screening identified 319 BC patients. The exclusion criteria for data collection were as follows: (a) bladder un-urothelial carcinoma confirmed by pathology (b) anti-tumor treatment was given at the time of diagnosis; (c) incomplete demographic information, clinicopathological features and follow-up data; (d) with concurrent malignant tumor or distant organ metastasis. In addition, those patients with certain diseases that may affect the level of AAPR, such as liver cirrhosis and hepatitis, were also excluded. The final cohort for further analysis therefore included 199 participants (**Figure 1**).

This study was approved by the Ethics Committee of Shengjing hospital of China Medical University. Since this is a retrospective cohort study, patients’ informed consent was not required.

### Data Collection and Variables

The clinicopathological and laboratory data used were collected from the hospital’s Electronic Medical Record system of a single-center database in a non-selective and continuous manner. The covariates included demographic and clinicopathological data, and variables that are known to possibly influence AAPR or clinical outcomes. Thus, the variables that were used to build the fully adjusted model were: age, sex, smoking history, Body Mass Index (BMI), Eastern Cooperative Oncology Group Performance Status (ECOG-PS), diabetes, American Society of Anesthesiologists (ASA), hypertension, chemotherapy, classification, T-stage, lymph node status, tumor grade and hemoglobin (HGB). Clinical staging of BC was based on the TNM staging criteria (American Joint Committee on Cancer [8th edition]). Histological grade was determined by postoperative pathological examination in accordance with the 2004 World Health Organization criteria. Other examinations included: routine laboratory tests, chest X-ray/CT, ultrasound, and bone scan for preliminary screening of distant metastases, and positron emission tomography (PET)-CT where necessary.

### Follow-Up

All patients were followed up regularly after surgery. Each patient underwent physical examination and laboratory testing every 3 months, and enhanced CT or MRI examination was performed every 3 to 6 months. If local recurrence or distant metastasis was suspected, CT, MRI, bone scan, pet-CT and other imaging examinations were immediately done. Overall survival (OS) was defined as the period from the operation to either death or last follow-up. Cancer-specific survival (CSS) was defined as the interval between surgery and bladder cancer‐related death. Recurrence-free survival (RFS) was defined as the time from surgery to first documentation of disease recurrence.

### Statistical Analysis

**Figure 2:**
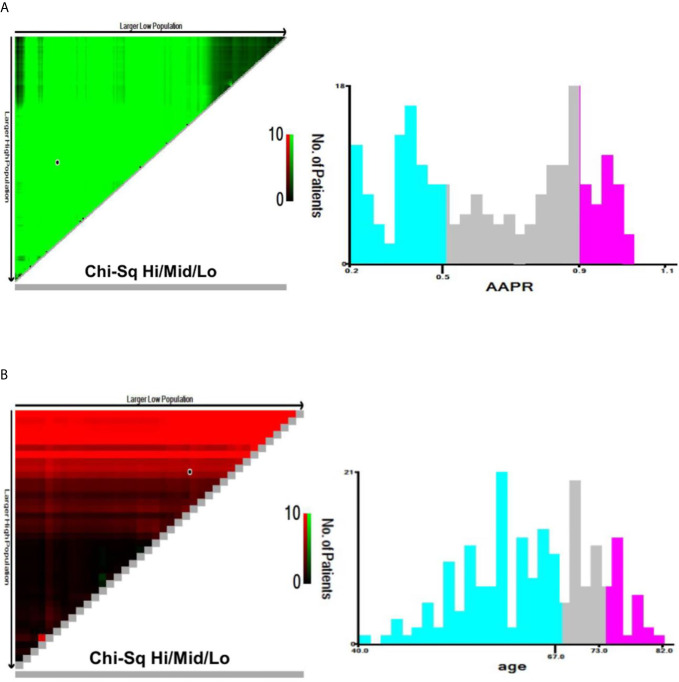
X-tile analyses of OS based on patient data to determine the optimal cut-off values for AAPR **(A)** and age **(B)**. The optimal cut-off values are shown in histograms of the entire cohort. OS, overall survival; AAPR, albumin-to-alkaline phosphatase ratio.

Continuous variables of normal distribution are represented as mean and standard deviation (SD), while those of skew distribution are shown as median and interquartile range (IQR). Categorical variables were expressed in frequency or as a percentage. AAPR was divided into three categories by X-tile software (version 3.6.1), which is a useful tool for finding optimal cutoff points of continuous data. The same method was adopted to identify appropriate cutoffs in age (**Figure 2**). Mann-Whitney U test and Chi-squared test was used to test for correlations between variables of different groups.

In this study, data analysis using hierarchical analysis and the adjustment of interference factors was undertaken in order to investigate the correlation between AAPR and clinical outcomes (linear or nonlinear), and to explore which factors affect this correlation. Therefore, we first established Cox proportional hazard models for univariate and multivariate analysis. We included the covariates into the Cox regression model and evaluated the influence of confounding factors by comparing the regression coefficients. The three models were: model 1, no adjustment of covariates; model 2, adjustment of social population data only; model 3, adjustment of all covariates. Linear regression was used for trend test. Next, we conducted a smooth curve fitting (penalty spline method) in order to study whether a nonlinear relationship existed between AAPR and OS. Subsequently, we constructed a hierarchical Cox proportional hazards model for subgroup analysis and likelihood ratio test was used to calculate the interaction between subgroups. The impact of AAPR on oncologic outcomes was evaluated by Kaplan-Meier curves (using log rank test). Furthermore, based on the multivariate Cox regression analysis, 3-year and 5-year nomograms of OS, CSS and RFS were constructed. The discriminative abilities of these nomograms were assessed by the concordance index (C-index) and the area under the curve (AUC). The calibration plot of the nomogram was evaluated by generating a calibration diagram by bootstrapping with 1000 resamples for the 3- and 5-year survival time. Analyses were performed using R software v.4.0.2 (http://www.R-project.org). All tests were two-tailed. Statistical significance was set at *P* < 0.05.

## Results

### Baseline Clinicopathologic Characteristics

Based on the cut-off value of AAPR calculated by the X-tile software, patients were divided into three groups: low-AAPR group (AAPR < 0.37, n = 35), medium-AAPR group (AAPR = 0.37-0.59, n = 61) and high-AAPR group (AAPR > 0.59, n = 103). The detailed characteristics based on AAPR groups were shown in **Table 1**. More patients with a lower BMI and history of smoking were found in the low AAPR group, with *P* value <0.05. However, no significant differences were detected in age (as contiguous and dichotomous variables), sex, smoking history, ECOG-PS, diabetes, hypertension, chemotherapy, ASA classification, T-stage, lymph node status, tumor grade and HGB among different AAPR groups (all *P* > 0.05).

**Table 1:** The relationship between AAPR groups and clinicopathological parameters in the present BC cohort (n = 199).

Covariates	Total	AAPR group			*P*-value
		Low	Medium	High	
Number of patients	199	35	61	103	
Age, Mean ± SD	64.0 ± 8.7	65.0 ± 9.2	65.1 ± 8.2	62.9 ± 8.8	0.227
Age group, n (%)					0.398
<67	124 (62.3)	18 (51.4)	36 (59)	70 (68)	
67-73	51 (25.6)	11 (31.4)	18 (29.5)	22 (21.4)	
≥74	24 (12.1)	6 (17.1)	7 (11.5)	11 (10.7)	
Sex, n (%)					0.591
Female	40 (20.1)	5 (14.3)	14 (23)	21 (20.4)	
Male	159 (79.9)	30 (85.7)	47 (77)	82 (79.6)	
BMI, Mean ± SD	23.5 ± 3.9	21.8 ± 3.6	24.5 ± 3.9	23.6 ± 3.9	0.005*
Smoking history, n (%)					0.038*
No	111 (55.8)	20 (57.1)	26 (42.6)	65 (63.1)	
Yes	88 (44.2)	15 (42.9)	35 (57.4)	38 (36.9)	
ECOG-PS, n (%)					0.505
0	134 (67.3)	26 (74.3)	42 (68.9)	66 (64.1)	
1	43 (21.6)	6 (17.1)	10 (16.4)	27 (26.2)	
2	22 (11.1)	3 (8.6)	9 (14.8)	10 (9.7)	
Diabetes, n (%)					0.412
No	164 (82.4)	30 (85.7)	47 (77)	87 (84.5)	
Yes	35 (17.6)	5 (14.3)	14 (23)	16 (15.5)	
Hypertension, n (%)					0.175
No	165 (82.9)	27 (77.1)	55 (90.2)	83 (80.6)	
Yes	34 (17.1)	8 (22.9)	6 (9.8)	20 (19.4)	
Chemotherapy, n (%)					0.282
No	164 (82.4)	32 (91.4)	50 (82)	82 (79.6)	
Yes	35 (17.6)	3 (8.6)	11 (18)	21 (20.4)	
ASA classification, n (%)					0.568
1	40 (20.1)	10 (28.6)	13 (21.3)	17 (16.5)	
2	130 (65.3)	20 (57.1)	38 (62.3)	72 (69.9)	
3	29 (14.6)	5 (14.3)	10 (16.4)	14 (13.6)	
T-stage, n (%)					0.249
T1-T2	166 (83.4)	26 (74.3)	51 (83.6)	89 (86.4)	
T3-T4	33 (16.6)	9 (25.7)	10 (16.4)	14 (13.6)	
Lymph node status, n (%)					0.15
Negative	160 (80.4)	24 (68.6)	51 (83.6)	85 (82.5)	0.586
Positive	39 (19.6)	11 (31.4)	10 (16.4)	18 (17.5)	
Tumor grade, n (%)					
Low	117 (58.8)	20 (57.1)	33 (54.1)	64 (62.1)	
High	82 (41.2)	15 (42.9)	28 (45.9)	39 (37.9)	
HGB, Median (IQR)	130.0 (116.0, 143.0)	127.0 (115.0, 138.0)	132.0 (112.0, 143.0)	131.0 (120.5, 143.0)	0.388
AAPR, Mean ± SD	0.6 ± 0.2	0.3 ± 0.1	0.5 ± 0.1	0.8 ± 0.1	< 0.001*

*P < 0.05. BC, bladder cancer; AAPR, albumin-to-alkaline phosphatase ratio; ECOG-PS, Performance Status of East Cooperative Oncology Group; ASA, American Society of Anesthesiologists; HGB, Hemoglobin.

The mean follow-up time was 24.0 months. At the last follow-up, 62 patients died of all-causes and 53 patients died of BC. Recurrence or metastasis occurred in 60 patients during follow-up. The 5-year OS, CSS, and RFS were 43.8%, 50.0%, 44.1% in patients with low-AAPR, respectively.

### Univariate and Multivariate Cox Regression Analysis

The association between selected clinicopathological features and OS, CSS and RFS was analyzed using univariable and multivariable Cox regression. In univariate Cox proportional hazards model, smoking history, high T-stage, high tumor grade, positive lymph nodes and medium/high AAPR were correlated with unfavorable OS. Whereas smoking history, ECOG-PS, high T-stage, high tumor grade, positive lymph nodes and medium/high AAPR were correlated with unfavorable CSS and RFS. We sequentially incorporated these variables into the multivariate Cox regression model and ultimately identified that smoking history, high T-stage, high tumor grade and medium/high AAPR were the independent prognostic factors influencing clinical outcomes (**Table 2**).

**Table 2:** Prognostic factors for OS, CSS and RFS of BC patients treated with RC in univariate and multivariate Cox regression analyses.

Covariates	OS				CSS				RFS			
	Univariate		Multivariate		Univariate		Multivariate		Univariate		Multivariate	
	HR (95%CI)	*P*-value	HR (95%CI)	*P*-value	HR (95%CI)	*P*-value	HR (95%CI)	*P*-value	HR (95%CI)	*P*-value	HR (95%CI)	*P*-value
Age	1.03 (1,1.06)	0.094			1.01 (0.98,1.05)	0.407			1.01 (0.98,1.04)	0.551		
Sex												
Female	1 (reference)				1 (reference)				1 (reference)			
Male	0.89 (0.48,1.65)	0.716			0.73 (0.39,1.36)	0.321			0.8 (0.44,1.46)	0.476		
BMI	1.47 (0.88,2.45)	0.140			1.02 (0.96,1.09)	0.526			1.03 (0.96,1.09)	0.427		
Smoking history												
No	1 (reference)				1 (reference)				1 (reference)			
Yes	2.1 (1.25,3.53)	0.005*	1.81 (1.0,3.29)	0.050*	3.46 (1.87,6.38)	< 0.001*	3.28 (1.60,6.72)	0.001*	3.04 (1.75,5.28)	< 0.001*	3.13 (1.63,5.98)	< 0.001*
ECOG-PS												
0	1 (reference)				1 (reference)				1 (reference)			
1	0.5 (0.24,1.02)	0.057			0.29 (0.11,0.74)	0.010*	0.44 (0.16,1.22)	0.114	0.43 (0.2,0.92)	0.030*	0.54 (0.23,1.27)	0.158
2	0.93 (0.44,1.99)	0.857			0.76 (0.32,1.79)	0.524	1.22 (0.44,3.41)	0.701	0.88 (0.41,1.87)	0.737		
Diabetes												
No	1 (reference)				1 (reference)				1 (reference)			
Yes	0.67 (0.32,1.36)	0.266			0.68 (0.32,1.47)	0.326			0.85 (0.43,1.69)	0.648		
Hypertension												
No	1 (reference)				1 (reference)				1 (reference)			
Yes	0.71 (0.35,1.45)	0.354			0.74 (0.35,1.57)	0.433			0.84 (0.42,1.65)	0.610		
Chemotherapy												
No	1 (reference)				1 (reference)				1 (reference)			
Yes	0.47 (0.21,1.03)	0.060			0.55 (0.25,1.24)	0.150			0.59 (0.28,1.24)	0.162		
ASA classification												
1	1 (reference)				1 (reference)				1 (reference)			
2	1.16 (0.59,2.27)	0.664			1.07 (0.52,2.18)	0.855			1.13 (0.58,2.22)	0.711		
3	1.31 (0.56,3.11)	0.533			1.32 (0.53,3.26)	0.550			1.1 (0.46,2.65)	0.834		
T-stage												
T1-T2	1 (reference)				1 (reference)				1 (reference)			
T3-T4	2.25 (1.28,3.95)	0.005*	2.43 (1.30, 4.56)	0.006*	2.59 (1.43,4.68)	0.002*	3.99 (1.94, 8.18)	< 0.001*	2.49 (1.41,4.37)	0.002*	3.88 (2.00, 7.55)	< 0.001*
Lymph node status												
Negative	1 (reference)				1 (reference)				1 (reference)			
Positive	1.76 (1.02,3.06)	0.044*	1.13 (0.60, 2.14)	0.696	2.07 (1.16,3.7)	0.014*	1.39 (0.70, 2.77)	0.350	1.85 (1.06,3.21)	0.029*	1.22 (0.64, 2.32)	0.540
Tumor grade												
Low	1 (reference)				1 (reference)				1 (reference)			
High	2.19 (1.31,3.65)	0.003*	2.27 (1.28, 4.03)	0.005*	2.92 (1.65,5.2)	< 0.001*	3.41 (1.71, 6.79)	0.001*	3 (1.76,5.12)	< 0.001*	3.47 (1.87, 6.44)	< 0.001*
HGB												
<130	1 (reference)				1 (reference)				1 (reference)			
≥130	1.01 (0.99,1.02)	0.331			1.19 (0.69,2.06)	0.539			1.08 (0.65,1.8)	0.763		
AAPR												
Low	1 (reference)				1 (reference)				1 (reference)			
Medium	0.54 (0.29,0.99)	0.048*	0.36 (0.17,0.77)	0.008*	0.52 (0.27,1)	0.051	0.32 (0.13,0.74)	0.008*	0.52 (0.28,0.96)	0.037*	0.29 (0.13,0.63)	0.002*
High	0.24 (0.13,0.46)	< 0.001*	0.21 (0.10,0.42)	< 0.001*	0.21 (0.11,0.42)	< 0.001*	0.18 (0.08,0.39)	< 0.001*	0.21 (0.11,0.41)	< 0.001*	0.17 (0.09,0.36)	< 0.001*

*P < 0.05. OS, overall survival; CSS, cancer-specific survival; RFS, recurrence-free survival; BC, bladder cancer; RC, radical cystectomy; AAPR, albumin-to-alkaline phosphatase ratio; ECOG-PS, Performance Status of East Cooperative Oncology Group; ASA, American Society of Anesthesiologists; HGB, Hemoglobin.

### Nonlinear Relationship Exploration

**Figure 3:**
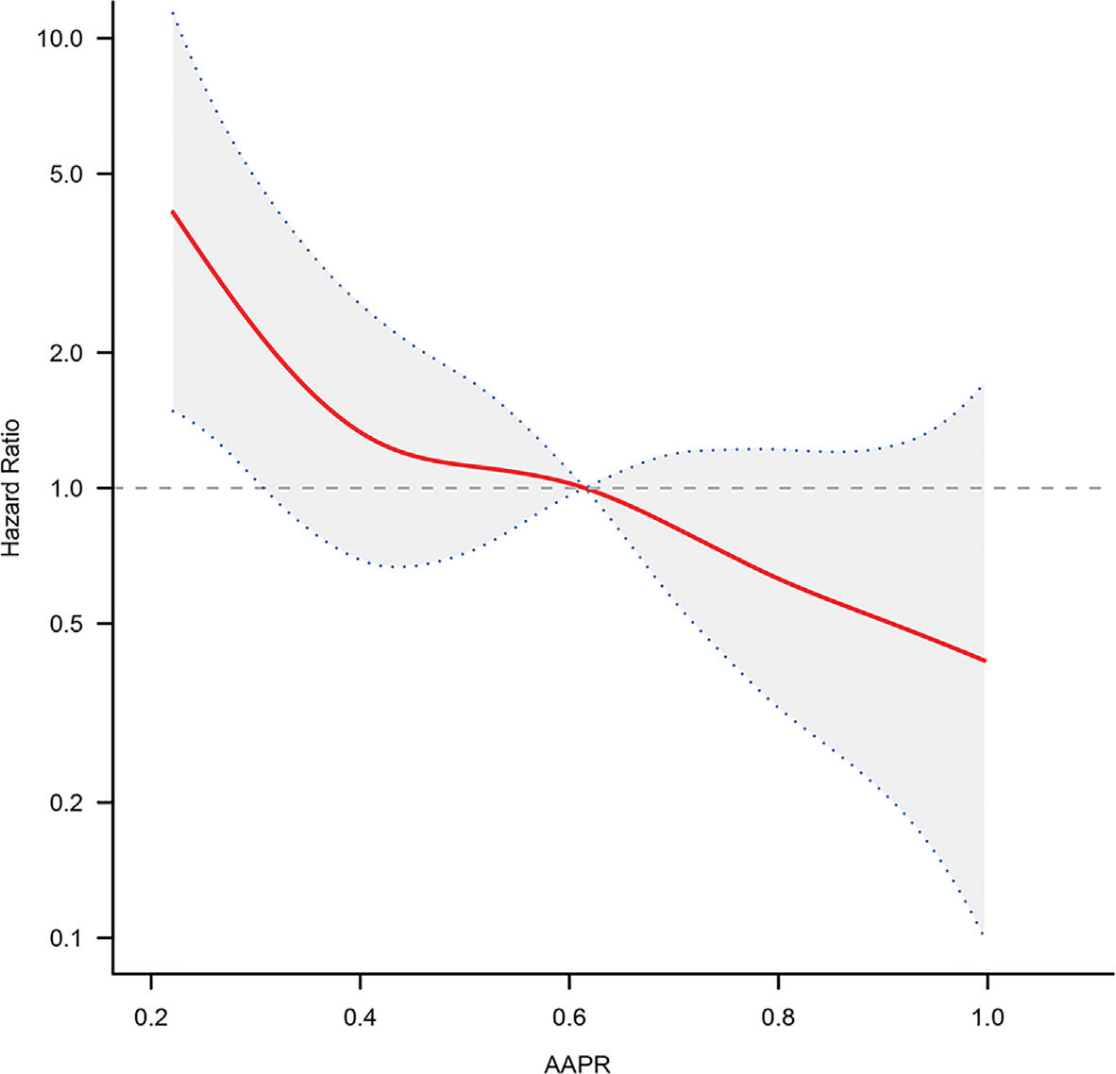
The adjusted smooth fitting curve between pretreatment AAPR and OS of BC patients based on two-piece-wise regression model. A linear relationship between AAPR and OS was observed. The red solid line and blue dashed line represent the estimated values and their corresponding 95% confidence intervals. OS, overall survival; AAPR, albumin-to-alkaline phosphatase ratio; BC, bladder cancer.

We analyzed whether there was a nonlinear relationship between AAPR and adverse outcomes (**Figure 3**). The smooth curve showed a linear correlation between AAPR and adverse outcomes after adjusting for possible confounding factors. The inflection point of 0.376 was found by the two-piecewise Cox proportional hazard model and recursive algorithm. The HR (95% CI) on left and the right side of the inflection point was 0.05 (0.00-0.72) (P = 0.0256) and 0.54 (0.16-1.65) (P = 0.3642), respectively. If taking 0.376 as the cut-off value, no saturation effect or threshold effect was found, with *P* for the likelihood ratio test >0.05.

### Construction of Unadjusted and Adjusted Cox Proportional Hazards Models

**Figure 4:**
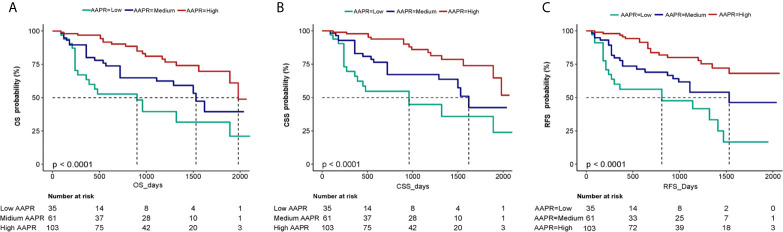
Kaplan-Meier curves of OS **(A)**, CSS **(B)** and RFS **(C)** in BC patients stratified by AAPR. OS, overall survival; CSS, cancer-specific survival; RFS, recurrence-free survival; BC, bladder cancer; AAPR, albumin-to-alkaline phosphatase ratio.

The independent impact of AAPR on OS was identified by three models (**Table 3**). As shown in the non-adjusted model, AAPR showed a negative correlation with the increasing risk for death (HR = 0.49, 95% CI = 0.36- 0.67). The HR of OS in the fully adjusted model was 0.44 and 0.25, respectively, which meant that, compared with the low AAPR group, the risk of death in the medium and high AAPR group was reduced by 56% (HR = 0.44, 95% CI = 0.21-0.91) and 75% (HR = 0.25, 95% CI = 0.12-0.49), respectively (all the *P* for trend <0.001). Kaplan-Meier curves showed significant associations of lower AAPR group with worse OS, CSS and RFS (**Figure 4**). The median OS of the low, medium, and high AAPR group was 12.5, 24, and 29 months, respectively (*P* value <0.0001).

**Table 3:** Multiple Cox regression analysis of AAPR in patients with BC.

AAPR	Non-adjusted	*P*-value	Adjust I	*P*-value	Adjust II	*P*-value
Continuous	0.1 (0.03,0.33)	< 0.001*	0.1 (0.03,0.33)	< 0.001*	0.09 (0.03,0.29)	< 0.001*
Group						
Low	1 (reference)		1 (reference)		1 (reference)	
Medium	0.54 (0.29,0.99)	0.048*	0.46 (0.24,0.88)	0.02*	0.44 (0.21,0.91)	0.027*
High	0.24 (0.13,0.46)	< 0.001*	0.22 (0.11,0.43)	< 0.001*	0.25 (0.12,0.49)	< 0.001*
*P* trend	0.49 (0.36,0.67)	< 0.001*	0.47 (0.34,0.66)	< 0.001*	0.5 (0.35,0.71)	< 0.001*

*P < 0.05. Non-adjusted model adjusted for: None. Adjust I model adjusted for: age, sex, BMI, ECOG-PS. Adjust II model adjusted for: age, sex, BMI, ECOG-PS, smoking history, diabetes, hypertension, chemotherapy, ASA, T-stage, lymph node status, grade and HGB.

### Subgroup Analysis

**Figure 5:**
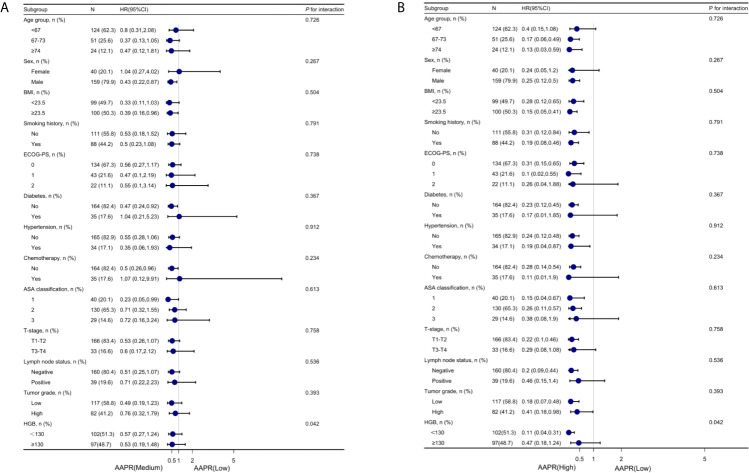
Forest plot for presenting the association between the hazard ratio of overall survival and medium- **(A)** and high-AAPR **(B)** in BC patients. AAPR, albumin-to-alkaline phosphatase ratio; BC, bladder cancer.

To further understand the negative correlation between AAPR and OS, we used the potential covariates listed in **Table 1** for stratified analyses. Age, sex, BMI, diabetes, hypertension, chemotherapy, ASA classification, ECOG-PS, T-stage, lymph node status, tumor grade, smoking history, and HGB were all stratified (**Table 4**). The trend of HRs in middle and high AAPR group decreased after stratification across all subgroups. As shown in **Figures 5A, B**, the results of subgroup analysis were presented visually by forest plots.

**Table 4:** Subgroup analysis using potential confounders as the stratification variables.

Confounding factor category	N	AAPR group			*P* for trend	*P* for interaction
		Low	Medium	High		
Age group, n (%)						0.726
<67	124 (62.3)	1 (reference)	0.8 (0.31,2.08)	0.4 (0.15,1.08)	0.052	
67-73	51 (25.6)	1 (reference)	0.37 (0.13,1.05)	0.17 (0.06,0.49)	0.001*	
≥74	24 (12.1)	1 (reference)	0.47 (0.12,1.81)	0.13 (0.03,0.59)	0.007*	
Sex, n (%)						0.267
Female	40 (20.1)	1 (reference)	1.04 (0.27,4.02)	0.24 (0.05,1.2)	0.04*	
Male	159 (79.9)	1 (reference)	0.43 (0.22,0.87)	0.25 (0.12,0.5)	< 0.001*	
BMI, n (%)						0.504
<23.5	99 (49.7)	1 (reference)	0.33 (0.11,1.03)	0.28 (0.12,0.65)	0.004*	
≥23.5	100 (50.3)	1 (reference)	0.39 (0.16,0.96)	0.15 (0.05,0.41)	< 0.001*	
Smoking history, n (%)						0.791
No	111 (55.8)	1 (reference)	0.53 (0.18,1.52)	0.31 (0.12,0.84)	0.023*	
Yes	88 (44.2)	1 (reference)	0.5 (0.23,1.08)	0.19 (0.08,0.46)	< 0.001*	
ECOG-PS, n (%)						0.738
0	134 (67.3)	1 (reference)	0.56 (0.27,1.17)	0.31 (0.15,0.65)	0.002*	
1	43 (21.6)	1 (reference)	0.47 (0.1,2.19)	0.1 (0.02,0.55)	0.006*	
2	22 (11.1)	1 (reference)	0.55 (0.1,3.14)	0.26 (0.04,1.88)	0.175	
Diabetes, n (%)						0.367
No	164 (82.4)	1 (reference)	0.47 (0.24,0.92)	0.23 (0.12,0.45)	< 0.001*	
Yes	35 (17.6)	1 (reference)	1.04 (0.21,5.23)	0.17 (0.01,1.85)	0.097	
Hypertension, n (%)						0.912
No	165 (82.9)	1 (reference)	0.55 (0.28,1.06)	0.24 (0.12,0.48)	< 0.001*	
Yes	34 (17.1)	1 (reference)	0.35 (0.06,1.93)	0.19 (0.04,0.87)	0.034*	
Chemotherapy, n (%)						0.234
No	164 (82.4)	1 (reference)	0.5 (0.26,0.96)	0.28 (0.14,0.54)	< 0.001*	
Yes	35 (17.6)	1 (reference)	1.07 (0.12,9.91)	0.11 (0.01,1.9)	0.034*	
ASA classification, n (%)						0.613
1	40 (20.1)	1 (reference)	0.23 (0.05,0.99)	0.15 (0.04,0.67)	0.016*	
2	130 (65.3)	1 (reference)	0.71 (0.32,1.55)	0.26 (0.11,0.57)	< 0.001*	
3	29 (14.6)	1 (reference)	0.72 (0.16,3.24)	0.38 (0.08,1.9)	0.227	
T-stage, n (%)						0.758
T1-T2	166 (83.4)	1 (reference)	0.53 (0.26,1.07)	0.22 (0.1,0.46)	< 0.001*	
T3-T4	33 (16.6)	1 (reference)	0.6 (0.17,2.12)	0.29 (0.08,1.08)	0.058	
Lymph node status, n (%)						0.536
Negative	160 (80.4)	1 (reference)	0.51 (0.25,1.07)	0.2 (0.09,0.44)	< 0.001*	
Positive	39 (19.6)	1 (reference)	0.71 (0.22,2.23)	0.46 (0.15,1.4)	0.171	
Tumor grade, n (%)						0.393
Low	117 (58.8)	1 (reference)	0.49 (0.19,1.23)	0.18 (0.07,0.48)	< 0.001*	
High	82 (41.2)	1 (reference)	0.76 (0.32,1.79)	0.41 (0.18,0.98)	0.033*	
HGB, n (%)						0.042*
<130	102(51.3)	1 (reference)	0.57 (0.27,1.24)	0.11 (0.04,0.31)	< 0.001*	
≥130	97(48.7)	1 (reference)	0.53 (0.19,1.48)	0.47 (0.18,1.24)	0.183	

*P < 0.05. AAPR, albumin-to-alkaline phosphatase ratio; ECOG-PS, Performance Status of East Cooperative Oncology Group; ASA, American Society of Anesthesiologists; HGB, Hemoglobin.

### Construction of Prognostic Nomograms

**Figure 6:**
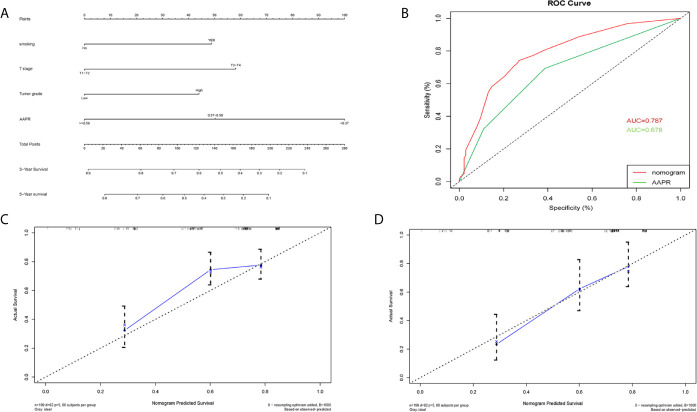
Nomogram model constructed by independent prognostic factors predicting 3- and 5-year OS for BC patients treated with RC **(A)**; the ROC curve of AAPR and AAPR-based nomogram for predicting OS in the primary cohort **(B)**; corresponding calibration curves of nomogram model for 3- and 5- year OS **(C, D)**. OS, overall survival; AAPR, albumin-to-alkaline phosphatase ratio; BC, bladder cancer; ROC, receiver operating characteristic; AUC, area under curve; CI, confidence interval.

**Figure 7:**
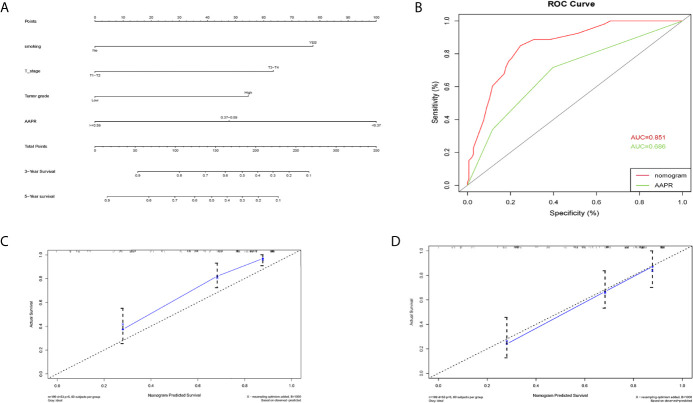
Nomogram model constructed by independent prognostic factors predicting 3- and 5-year CSS for BC patients treated with RC **(A)**; the ROC curve of AAPR and AAPR-based nomogram for predicting CSS in the primary cohort **(B)**; corresponding calibration curves of nomogram model for 3- and 5- year CSS **(C, D)**. CSS, cancer-specific survival; AAPR, albumin-to-alkaline phosphatase ratio; BC, bladder cancer; ROC, receiver operating characteristic; AUC, area under curve; CI, confidence interval.

**Figure 8:**
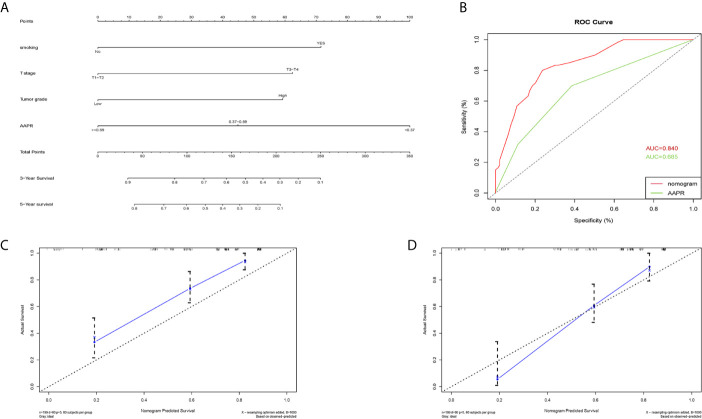
Nomogram model constructed by independent prognostic factors predicting 3- and 5-year RFS for BC patients treated with RC **(A)**; the ROC curve of AAPR and AAPR-based nomogram for predicting RFS in the primary cohort **(B)**; corresponding calibration curves of nomogram model for 3- and 5- year RFS **(C, D)**. RFS, recurrence-free survival; AAPR, albumin-to-alkaline phosphatase ratio; BC, bladder cancer; ROC, receiver operating characteristic; AUC, area under curve; CI, confidence interval.

A nomogram for 3- and 5- year OS was formulated based on the results of the multivariable Cox regression analyses (**Figure 6A**). The C-index of the nomogram was 0.728 (95% CI 0.663-0.793). ROC curves of AAPR and the AAPR-based nomogram for predicting OS are shown in **Figure 6B**, with the AUC 0.678 and 0.787, respectively. The calibration plot for predicting the OS of BC patients fitted very well between the nomogram-predicted probability and actual observation at 3- and 5- years after operation (**Figures 6C, D**). Moreover, the AAPR-based nomograms for CSS and RFS were also constructed (**Figures 7A–D** and **8A–D**), both showing consistency with the results of OS. The C-index showed a good predictive accuracy for nomograms in both CSS and RFS (CSS, C-index 0.792, 95% CI 0.748-0.838; RFS, C-index 0.784, 95% CI 0.739-0.829).

## Discussion

Previous studies have shown that the high invasiveness of BC is associated with high recurrence rate and low disease-specific survival rate (19, 20). Identifying those patients with a poor prognosis is therefore of great significance in BC treatment. A number of studies have confirmed that, in predicting the prognosis of several types of cancer, AAPR has shown promising results. However, its value in bladder cancer has not been reported so far. In the present study, we investigated the role of pretreatment AAPR as a valuable prognostic factor for BC patients who underwent RC, which may help to determine the optimal treatment.

To date, many prognostic indicators for BC, such as the TNM staging system and the histological grade, are mainly obtained from examination of tumor tissues after surgery. Therefore, due to the lack of effective biomarkers, it is difficult to evaluate the postoperative risk of BC patients before operation. Current studies show that not only the tumor itself but also other host-related factors contribute to tumor progression and patient prognosis (21, 22).

Earlier studies have shown that impaired nutritional status may be associated with cancer progression (23–25). As one of the main components of serum proteins, ALB can be used as a marker in monitoring nutritional status and systemic inflammation (26, 27). It also promotes DNA replication and cell growth, and plays an antioxidant role in tumorigenesis (28–30). Hypoalbuminemia has been shown to be associated with decreased survival of different types of cancer, including BC (31–33). Hypoproteinemia has also been reported to be closely related to the progression of bladder cancer (34, 35). Data from Peng et al. has shown that a preoperative low ALB level in patients with BC resulted in an increased risk of overall and cancer-specific death, compared with patients with a normal ALB level, and that the decreased serum ALB level may be a risk factor for poor tumor prognosis (36).

ALP is a specific phosphatase, which can dephosphorylate various types of molecules (such as nucleotides, proteins and alkaloids). Studies have shown ALP to be a marker for the prognosis of esophageal cancer (37), nasopharyngeal carcinoma (38), and renal cell carcinoma (39). Different levels of ALP expression in BC tissues can predict the risk of bone metastasis, and, in the follow-up of patients, determine whether the occurrence of bone metastasis is likely (40). Therefore, its potential value as a prognostic marker in cancer patients should be explored.

Serum AAPR is a comprehensive reflection of a host’s nutritional status and provides important information about their condition, which facilitates prognostic evaluation. AAPR was first proposed as a prognostic marker for hepatocellular carcinoma by Chan et al. (15), who proved that it was independently related to the clinical prognosis of patients with hepatocellular carcinoma undergoing radical surgery. Tan et al. (41) subsequently showed that low AAPR (AAPR < 0.58) was an independent prognostic factor for OS (HR = 1.587, 95% CI 1.185 - 2.126, P = 0.002) and PFS (HR = 1.337, 95% CI 1.027 - 1.739, P = 0.031) in patients with upper urothelial carcinoma undergoing radical surgery. Moreover, Xia et al. (17) showed that low AAPR (AAPR < 0.39) was an independent prognostic factor for OS (HR = 2.745, 95% CI 1.26 - 5.953, P = 0.01) and tumor specific survival (HR = 3.042, 95% CI 1.278 - 7.243, P = 0.012) in patients with non-metastatic RCC undergoing radical nephrectomy. In our study, the results showed that a lower pretreatment AAPR was associated with worse clinical outcome in BC patients receiving RC. The survival curve showed that there were significant differences in survival rate among the three groups (P < 0.001). Similarly, we found that in the fully adjusted model, the risk of postoperative death decreased and OS prolonged with the increase of AAPR. Subgroup analysis also confirmed the robustness and repeatability of these results. The relationship between AAPR and clinical outcomes did not seem to be nonlinear, and no significant inflection point was found in smooth curve fitting. Therefore, we used the X-tile software to determine the cut-off value. It should be noted that the optimal cut-off value of AAPR in predicting tumor outcome may be different among different cancer types, which may be due to tumor specificity, or due to different sample size, follow-up time and survival endpoint.

Recently, the nomogram has been widely used to predict the prognosis of various cancers (42–44). In our present study, novel nomograms were first developed in BC patients treated with RC using clinicopathological factors including AAPR. We used the independent prognostic factors in multivariate analysis to construct - nomograms to predict the 3-year and 5-year OS, CSS and RFS of patients with bladder cancer clearly and effectively. Using this nomogram, we can predict the outcome of individual patients, thus bringing benefits to clinicians and patients. The nomograms provided relatively high C-index and AUC for OS, CSS and RFS rates., showing the accuracy of prognosis prediction. The calibration curves also showed the consistency between the prediction probability of the model and the observation results.

Our research has several strengths. Firstly, to the best of our knowledge, we are the first to explore the clinical outcomes of AAPR and BC patients treated with RC, and to establish a valuable predictive nomogram for predicting the postoperative survival of these patients. The findings of this study will contribute to the establishment predictive models for these patients in the future. Determination of AAPR is helpful for screening high-risk patients with BC and guiding treatment after RC. The AAPR-based pre-treatment nomogram is helpful to identify BC patients with poor prognosis, so as to carry out effective treatment earlier to prevent adverse outcomes. For high-risk patients, a more thorough and frequent follow-up may be required. If necessary, PD-1 or other immunotherapy after surgery can be used in high-risk BC patients. Moreover, since patients with low-AAPR were more likely to have a poor prognosis with the possibility of impaired immune-nutritional status and inadequate response to the surgical stress, these patients need to increase nutrition intake to improve albumin level and systematic immunity during their treatment, which may play a key role in their prognosis. Secondly, in order to reduce bias, we adjusted confounding factors to explore the correlation between AAPR and clinical outcomes more accurately and ensure the authenticity and reliability of observational research results. Thirdly, subgroup analysis was used to evaluate the consistency and robustness of the results among subgroups of patients. Finally, covariates that can be easily identified in clinical practice, such as ALB and ALP, were assessed. Indeed, albumin and alkaline phosphatase levels are routinely tested when patients are admitted and protein testing is a low-cost, highly efficient, and commonly available preoperative indicator.

However, there are some limitations to our study. Firstly, it was a retrospective observational study conducted in a single medical institution in China, and the scope of our database was not large, so selection bias may exist. Secondly, although the Cox regression model was used to adjust the confounding factors, there may be many other clinicopathological factors affecting AAPR, which could not be completely excluded. Thirdly, it is unclear whether the clinical application of AAPR can be extended to all patients with BC. Fourthly, the sample size was relatively small, so the statistical significance of differences in some parameters may be slightly distorted and the nomogram may be slightly underpowered. In addition, although the nomograms showed good C-indexes, they have not been validated in an independent cohort. Therefore, further validation of the ability and accuracy of AAPR to predict the clinical prognosis of BC patients warrants larger, multicenter, prospective studies.

## Conclusions

AAPR is a novel and easily available clinical indicator related to poor prognosis in BC patients who underwent RC. Our study indicates that it has the potential to be served as an independent prognostic factor, moreover, the preoperative AAPR-based nomograms also have predictive value for the prognosis of patients with BC treated with RC. However, the clinical application of AAPR still needs to be confirmed by multi-center, prospective analysis.

## Data Availability Statement

The raw data supporting the conclusions of this article will be made available by the authors, without undue reservation.

## Ethics Statement

The studies involving human participants were reviewed and approved by The Ethics Committee of Shengjing hospital of China Medical University. Written informed consent for participation was not required for this study in accordance with the national legislation and the institutional requirements.

## Author Contributions

XC and SjL conceived and designed the study. SjL and SyL contributed to the data collection and analyses of the data. SjL drafted and revised the manuscript. SjL, SyL, and XL prepared figures and/or tables. XL and XC edited the manuscript. All authors contributed to the article and approved the submitted version.

## Funding

This study was supported by the Joint Plan of Key Research and Development Program of Liaoning Province (Grant No. 2020JH 2/10300137), Joint Plan of Key Research and Development Program of Liaoning Province (Grant No. 2020JH 2/10300148), and the 345 Talent Project of Shengjing Hospital (Grant No. M0716).

## Conflict of Interest

The authors declare that the research was conducted in the absence of any commercial or financial relationships that could be construed as a potential conflict of interest.
